# Development of a novel approach to enhance the solubility of ftibamzone formulation

**DOI:** 10.15761/IMM.1000111

**Published:** 2014-11-07

**Authors:** Ofonime Udofot, Kristen Jaruszewski, Shawn Spencer, Edward Agyare

**Affiliations:** 1 Division of Basic Pharmaceutical Sciences, College of Pharmacy and Pharmaceutical Sciences, Florida A and M University, Tallahassee, FL, USA; 2 The University Texas, MD Anderson Cancer Center, Houston, TX, USA

**Keywords:** ftibamzone, genital herpes, nanoparticles, simplex herpes virus, solubility

## Abstract

Ftibamzone (FBZ) is known to be effective against herpes simplex virus that causes genital herpes but poor solubility of FBZ has reduced its therapeutic efficacy. We investigated water-soluble complexes of various nanoparticles with FBZ to improve its solubility as well as increase its absorption. Using phase-solubility technique, we measured formation constant (K_1:1_ and K_1:2_) values at room temperature in pH 7 buffer. Solubility was determined by dissolving FBZ or FBZ-entrapped nanoparticles in phosphate buffers and pH adjusted to different pH range (2–12). The solutions were then equilibrated for 24 hours and then filtered and analyzed using HPCL. Nanoparticles were formulated using nanoprecipitation technique and cellular uptake of nanoparticle was determined by confocal microscope. No significant FBZ solubility was observed from pH 2 to 10 however we did notice a rapid increase in solubility from pH of 10 to 12 with FBZ solubility of 950 μg/ml. Our log D against pH profile revealed that FBZ is characteristic of an acid drug since unionized group was dominant at low pH. FBZ interaction with methyl-β-cyclodextrin (mβCD) complexation/nanoparticles showed a greater solubility of FBZ compared with FBZ alone while complexation constants were determined to be K_1:1_ and K_1:2_ were 7.06×10^−3^ and 8.98×10^−8^ mM^−1^ respectively. Only FBZ-chitosan nanoparticles were toxic against MDCK cells. Study demonstrates that FBZ-PLGA nanoparticles could significantly enhance the solubility and absorption of FBZ compared with FBZ alone and has the potential to be used as an effective delivery system for the treatment of genital herpes.

## Introduction

Genital Herpes is a sexually transmitted disease (STD) caused by the herpes simplex viruses which could either be type 1 (HSV-1) or type 2 (HSV-2) [1–5]. Majority of genital herpes cases are caused by the type 2 (HSV-2). Most individuals infected with HSV show or exhibit no signs or symptoms from HSV-1 or HSV-2 infection [2] and when signs do occur, they typically appear as one or more blisters around the genitals/rectum and mouth. The blisters break leaving tender ulcers (sores) that may take two to four weeks to heal the first time they occur. Typically, a second outbreak can appear weeks or months after the first, but it is always less severe and for shorter period of time compared to the first outbreak. Genital herpes is mostly contracted through sexual contact with an infected individual. Those infected with genital herpes do not show any signs or symptoms until they have an outbreak. Common symptoms associated with genital herpes could be systemic while some are localized. Systemic symptoms include pain, decreased appetite, fever and muscle ache. While localized symptoms which are usually confined to the genital regions include blisters, that are fluid filled, and take several days to heal. In women this localized symptom is always found on the outer labia of the vagina, around the anus, thighs and buttocks, while in men it is expressed on the penis, scrotum, thighs and buttocks. These symptoms may also be accompanied with vaginal discharge, painful urination and an enlarged lymph node. It is also important to note that when a person gets infected that the virus remains dormant in the body for a long period of time and recurrent infections and symptoms are milder in men than women. Genital herpes is clinically diagnosed by physical examination and performing laboratory test to confirm the presence of the virus. These laboratory exams include blood test, deoxyribonucleic acid (DNA) test and viral culture [6].

Currently, there are no available treatments that can cure genital herpes, but there are drugs which have the capability of suppressing the severity and duration of symptoms during outbreaks, hasten the time for lesion healing and cessation of viral shedding which ultimately helps in reducing the chance of infecting another person. These antivirals drugs include famciclovir, valacyclovir and acyclovir and related nucleotide analogs [2,5]. However emergence of resistance and toxicity to these drugs have rendered them ineffective for the treatment of HSV infected immunocompromised individuals and highlight the need for new, safe and effective antiviral drugs.

Ftibamzone, which belongs to thiosemicarbozones group, has been shown to possess an inhibitory effect on virus plaque formation and reproduction against HSV-1 and HSV-2. It has largely been used as ftibamzone cream or ointment for the treatment of HSV in Egypt and China [7–10], though its use has not been approved in the United States [11,12]. In addition, FBZ has a unique mechanism of antiviral action which is believed to be associated with ribonucleotide reductase inhibition compared to antiviral nucleotide analogues. But low activity and poor solubility of FBZ have reduced its therapeutic efficacy and limited its medicinal application [13] such as inability to be administered intravenously. We chose a polarized cell line, Madin-Darby canine kidney (MDCK) cell line for this study because the spectrum of cells infected by HSV in humans consists largely of polarized cells [14]. In this study we report that novel formulation of FBZ entrapped nanoparticles can improve FBZ solubility and increase absorption of FBZ by epithelial cells effectively treat genital herpes.

## Materials and methods

### Materials

All the chemicals and reagents were purchased from Sigma-Aldrich (St. Louis, Missouri, USA). Poly lactic-co-glycolic acid (PLGA) 50/50 (MW 153,000) was a generous gift from Purac Biomaterials (Gorinchem, Netherlands).

### Physiochemical characterization of FBZ

#### pH-Solubility profile

The aqueous solubility of FBZ in phosphate buffers with pH values ranging between 2 and 12 was determined. According to procedure described previously [15–17], excess FBZ was added to various vials containing 10 ml buffer, placed in a water-bath shaker at 37°C, and allowed to equilibrate for 24 hours. Following equilibration, the samples were centrifuged at 4,500 rpm for 5 minutes in a Thermo IEC Multi RF Centrifuge (Needham Heights, Massachusetts USA). The supernatants were collected, filtered through a 0.45 μm filter, and assayed for FBZ by HPLC. All experiments were performed in triplicates.

#### pKa-determination from FBZ-pH solubility profile

pKa values were determined from the pH-solubility profile based on the methods described previously [18,19]. From the equation provided:

(1) $$\mathrm{pKa}=pH\pm \log\left(\frac{S-S_{o}}{S_{o}}\right)$$

where S_o_ is the solubility of the unionized form of FBZ in water, and S is the observed solubility of drug at specified pH values, a plot of the $\log\left(\frac{S-S_{o}}{S_{o}}\right)$ versus pH was used to determine pKa from the x-axis intercept.

### Distribution coefficient

A 1-octanol/water partition coefficient was performed by placing 5 mg of FBZ in 5 mL phosphate buffer (0.1 M) with pH ranging from 2 to 10, that had been previously been saturated with 1-octanol. An equal volume of 1-octanol (5 mL), which served as the organic phase, was added to the aqueous phase. Tubes were rotated end-over-end for 5 hours and centrifuged for 10 minutes at 4,500 rpm. Finally the aqueous phase was carefully aspirated from the organic phase diluted, and amount of FBZ present was determined using UV/Vis spectrophotometry. The partition coefficient (Log P) was calculated using the equation:

(2) $$\log P=\frac{\mathrm{Vaq}(C_{i}-C_{aq})}{V_{o}C_{aq}}$$

Where *V_aq_* is the volume of the aqueous phase, *C_i_* and *C_aq_* are the concentration of FBZ in the aqueous phase initially and after equilibration and *V_o_* is the volume of 1-octanol.

### Ftibamzone stability

Forced degradation studies were conducted as by Ohja *et al.* [20]. One milligram of FBZ was dissolved in 1M hydrochloric acid at room temperature and sampled over a period of 120 minutes and assayed for intact FBZ by HPLC.

#### HPLC analysis of FBZ

The mobile phase consisting of 60% acetonitrile and 40% of 50 mM phosphate buffer with 1mM EDTA was pumped at a flow rate of 1.0 mL/min through a Zorbax 300SB-C18, 4.6×250 mm, 5 micron column (Agilent, Santa Clara CA) at room temperature. The sample injection volume was 20 μL and the FBZ was detected at 345 nm with SPD-M10AVP photodiode detector (Shimadzu, Columbia Maryland. USA). The data was collected and integrated using Shimadzu EZ Start 7.2.1

### Formulation of various nanoparticles loaded with FBZ

#### FBZ and methyl-β-cyclodextrin (mβCD) complexation

Solutions containing 1%, 2%, 5% and 10% *mβCD* were prepared in distilled water; FBZ was added to each solution, stirred for 48 hr and then centrifuged at 4,500 rpm for 10 minutes. The filtrate was obtained, by collecting the supernatant and subsequently filtering through a 0.45 μm filter [21,22]. The absorbances of the filtrates were obtained at various wavelengths using a SkanltMultiskan Spectrum spectrophotometer (Thermo Electron Corporation, Vantaa, Finland). The complexation constant (K_1:1_), according to the hypothesis of 1:1 stoichiometric ratio of complexes was calculated from phase solubility diagrams using the following equation:

(3) $$K_{1:1}=\frac{\mathrm{slope}}{So(1-\mathrm{Slope})}$$

Where, K_1:1_ is the complexation constant, S_o_ is the intrinsic solubility, and the slope is calculated from a graph of the dissolved drug concentration versus mβCD concentration in the medium. The intrinsic solubility of FBZ in the absence of mβCD was determined directly in aqueous media.

The 1:2 complex (K_1:2_) was calculated using an equation derived by Loftsson *et al.* below [23]:

(4) $$S_{t}=S_{o}+K_{1:1}.S_{o}.[\text{CD}]+K_{1:1}{\text{.K}}_{1:2}.S_{o}{\left[\text{CD}\right]}_{2}$$

Where S_t_ and CD is the total FBZ and mβCD concentration respectively, K_1:1_ and K_1:2_ represent the complexation constants for the 1:1 and 1:2 complexes respectively.

#### Chitosan gel containing FBZ/mβCD

Chitosan hydrogels were prepared by dissolving 400 or 800 mg chitosan in 20 ml of 1% glacial acetic acid solution. Twenty milliliters of 10% FBZ/mβCD complex was then added to each chitosan gel preparation and mixed thoroughly to form 1% and 2% chitosan gels containing FBZ/mβCD.

#### FBZ-PLGA nanoparticles preparation

Nanoparticles (FBZ-PLGA) were prepared using the nanoprecipitation technique previously described [24,25]. Poly (vinyl alcohol) (PVA) was employed in the formulation to increase drug uptake and release characteristics. Briefly, 1 mg of FBZ was added to 2 ml of 1% PLGA in acetone and stirred until dissolved and followed by the addition of 4% PVA solution in a drop-wise fashion in FBZ-PLGA under constant stirring at 1,500 rpm. The formed emulsion was stirred at room temperature for 2 hours to evaporate acetone. Larger particles and polymer clumps were removed by centrifuging the suspension at 5,000 rpm for 10 min. Blank PLGA nanoparticles (PLGAnps) without FBZ were also formulated similarly. The particle size and zeta potential of the all formulations (PLGAnps and FBZ-PLGAnps) were measured using BI-200SM laser light scattering system (Brookhaven Instruments, Holtsville, New York).

#### FBZ leakage from FBZ-PLGAnps

To assess the leakage of FBZ from PLGAnps, 3 ml nanoparticle suspension was transferred to a 50 kDa molecular weight cut off dialysis bag and dialyzed against 50 ml PBS. At predetermined intervals, 500 μl of dialysate was sampled and replaced with equal volume of fresh PBS buffer. The FBZ concentrations in the samples were determined by HPLC.

### Effect of various nanoparticles on polarized MDCK cells

Madin Darby Canine Kidney (MDCK) cells were seeded on 96 well plates at 5 × 10^3^ cells per well and cultured in DMEM supplemented with 2 mM L-glutamine, 1.5 g/L sodium bicarbonate, 4.5 g/L glucose, 10 mM HEPES, 1 mM sodium pyruvate, 10% fetal bovine serum, and 1% penicillin/streptomycin. At 75% confluence, the MDCK cells were exposed to various formulations (PLGAnps, FBZ-PLGAnps, FBZ-mβCD and FBZ-1% chitosan gel) and cell viability was determined using Alamar blue assay at 6, 24 and 48 hr time points. At the end of each time point, the cells were washed with HBSS and further incubated for 5 h with 100 μl of Alamar blue plus cell medium. The absorbance of the produced color was measured using Skanlt Multiskan Spectrum spectrophotometer (Thermo Electron Corporation, Vantaa, Finland) at a wavelength of 550/580 nm.

### Confocal Effect of various nanoparticle formulations on epithelial cells

Polarized MDCK cells were incubated with FBZ-PLGAnps conjugated-fluorescein isothiocyanate (FBZ-PLGAnps-FITC) at 37 °C with gentle shaking. After 1 hr, the FBZ-PLGAnps-FITC were removed, the cell surface was washed twice with PBS, fixed using 4% paraformaldehyde, mounted, and imaged with excitation wavelength of 488 nm and emission wavelength of 520 nm using Axiovert 100M microscope equipped with Zeiss LSM 510 laser confocal microscope (Carl Zeiss, Inc., Thornwood, NY).

## Results

### pH-solubility profile and pKa

**Figure 1:**
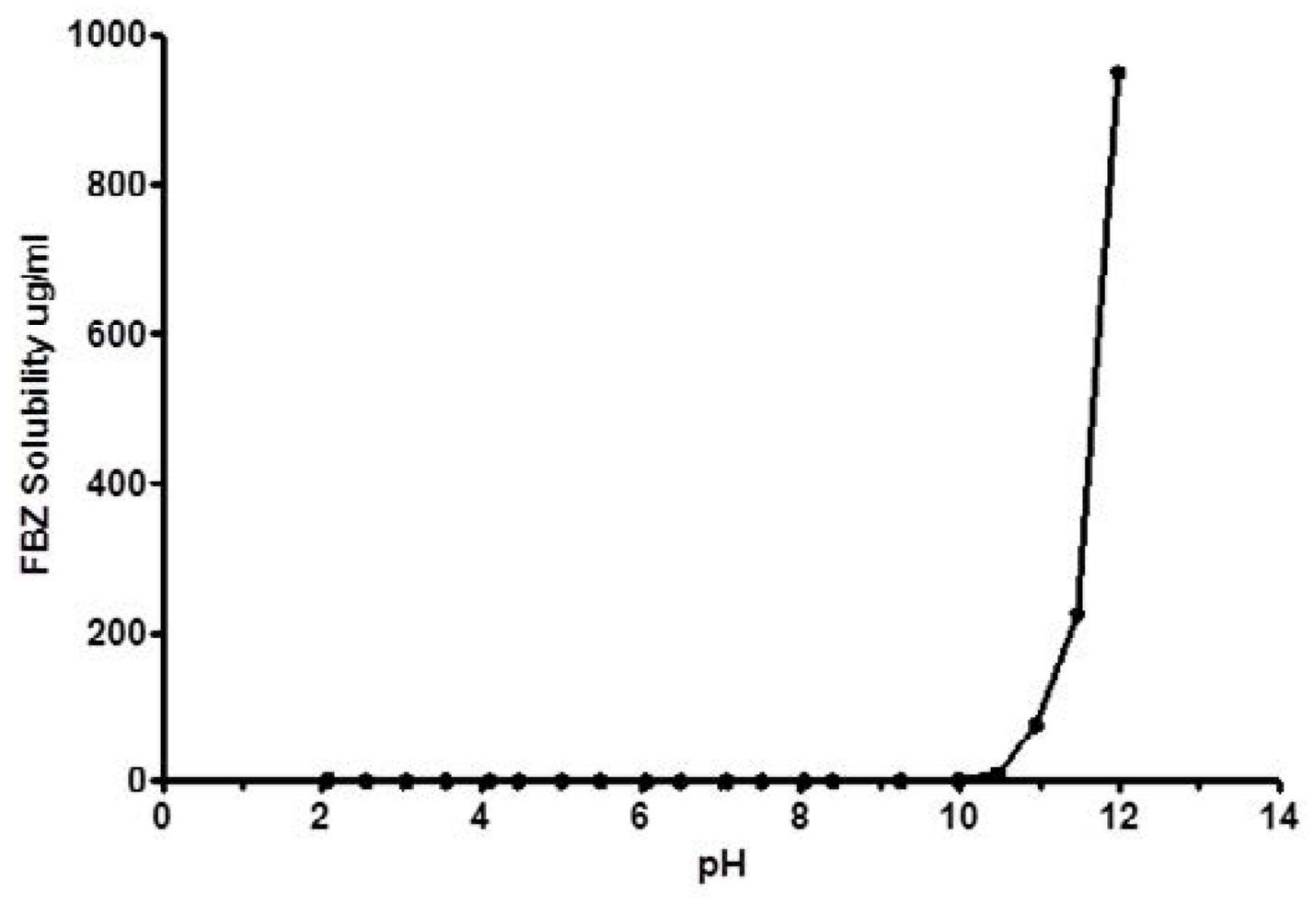
FBZ p*H*-solubility profile at 25 ± 1°C

**Figure 2:**
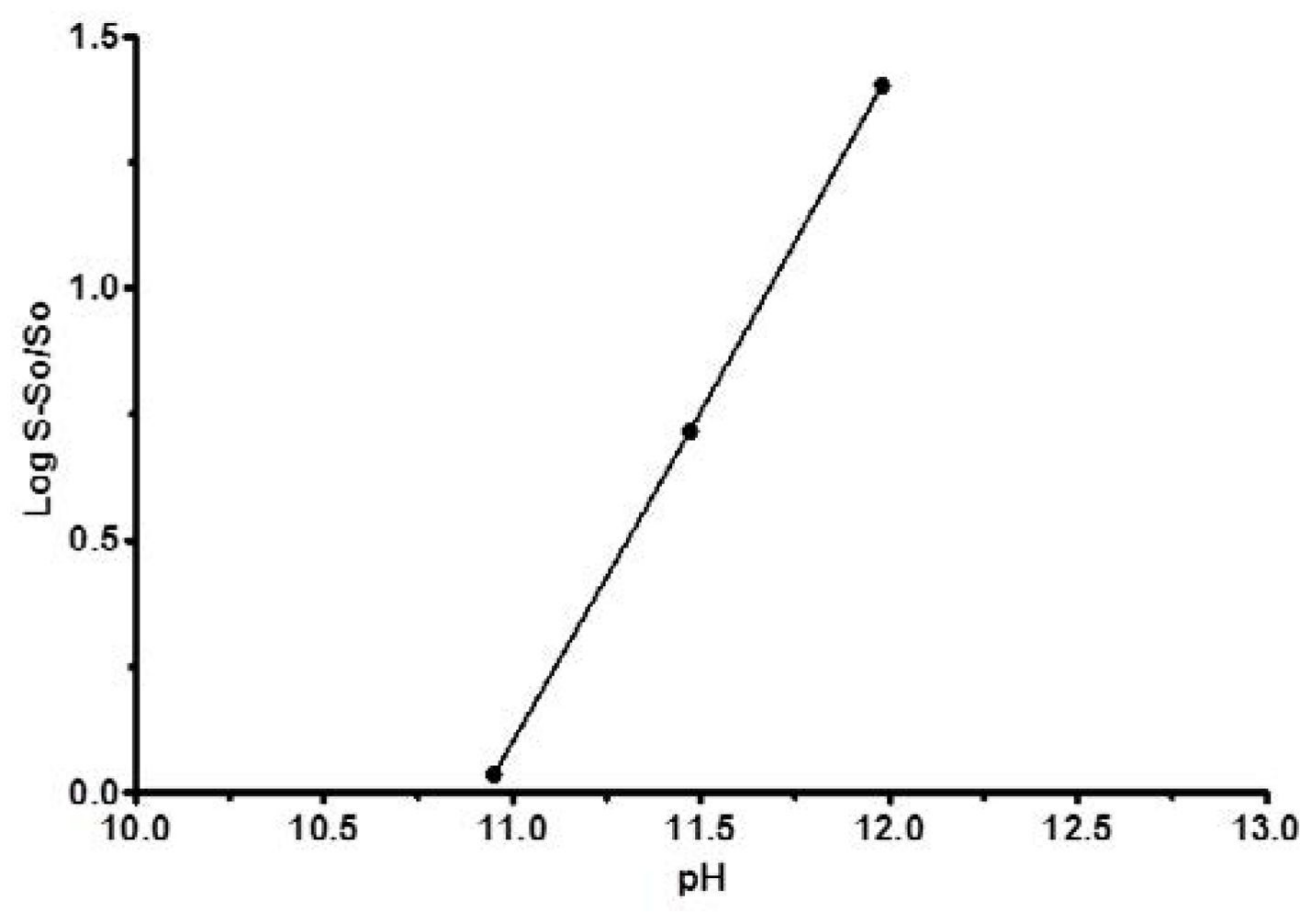
FBZ pKa as a function of solubility; a plot of $\mathrm{Log}\left(\frac{S-S_{o}}{S_{o}}\right)$ as a function of pH is shown. The plot was found to be linear with a R^2^ value >0.99. The intercept on the x-axis is the apparent pKa for FBZ.

No significant increase in FBZ solubility was observed from pH 2 to 10 as shown in Figure 1, however FBZ solubility increased significantly from pH 10 to pH 12 with a final solubility of 950 μg/ml. Incorporating pH-solubility data in equation 1, the pKa of FBZ was determined to be 10.95 (Figure 2) while the calculated log P using octanol/water (equation 2) was found to be 1.246.

### Log Distribution coefficient and stability

**Figure 3:**
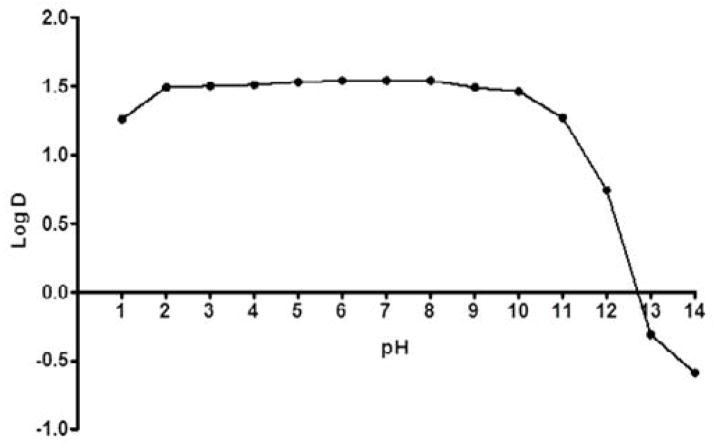
FBZ Distribution coefficient profile; the relationship between the partition coefficient and pH, for Ftibamzone in the octanol-buffer system.

**Figure 4:**
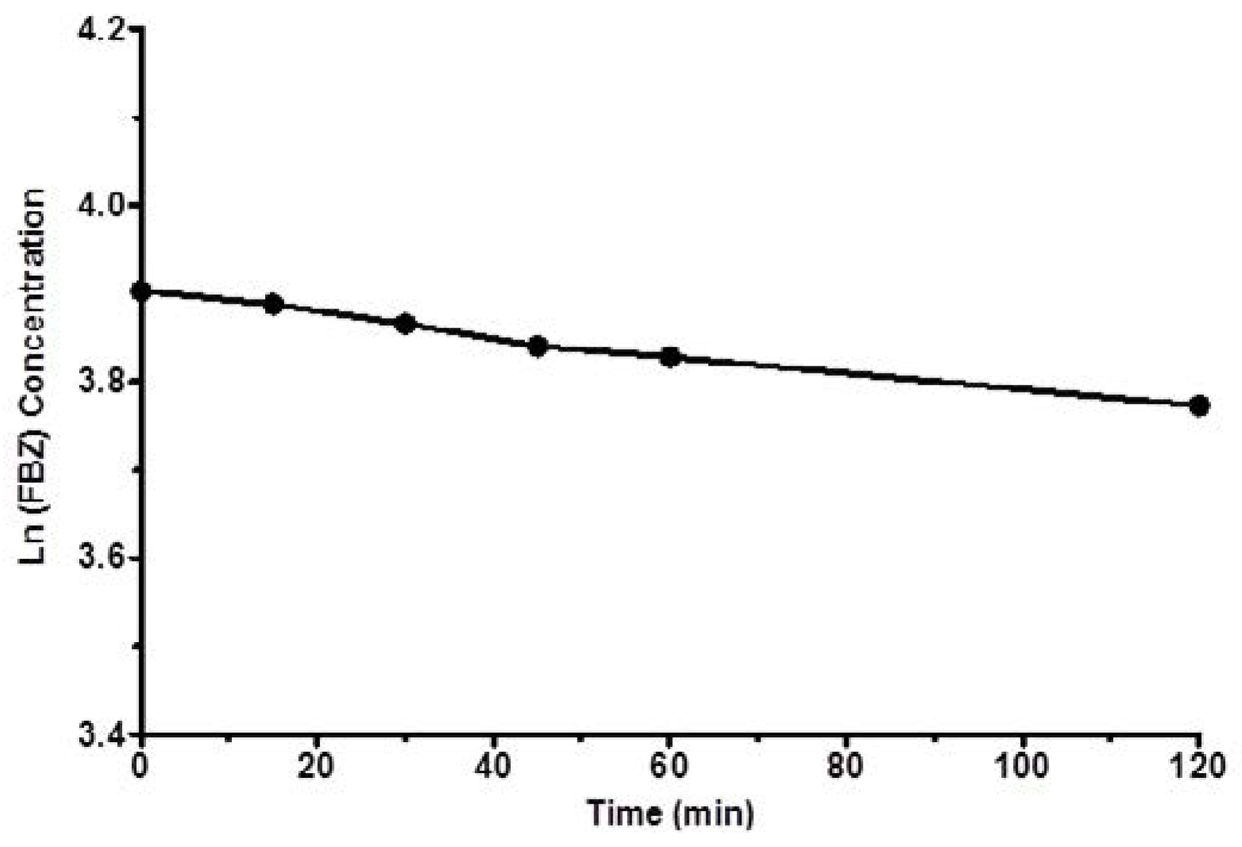
Degradation profile of FBZ in HCl versus time as shown over period of 120 min. Data are expressed as mean ± SEM (n=3).

Figure 3 shows log D versus pH profile of FBZ solubility at different pH points. From the profile, it is observed that the log D value increased slightly from 1.26 at pH 1 to 1.49 at pH 2 and thereafter remains unchanged to pH 10. Afterwards, log D decreased remarkably from 1.42 to −0.59 from pH range of 11 to 14. This suggests that log D against pH profile of drug FBZ is a characteristic of an acid drug where unionized group dominant at low pH. While at high pH, drug FBZ reaches ionized state and increases its hydrophilic nature. For the stability of FBZ in an acid environment, FBZ degradation was assessed in 1M HCL and the data obtained (Figure 4) revealed that over the period of 120 min, only 2.21% of FBZ was degraded. Overall, the stability profile of FBZ showed a degradation rate of 2.5×10^−5^ min^−1^.

### FBZ-mβCD complexation

**Figure 5:**
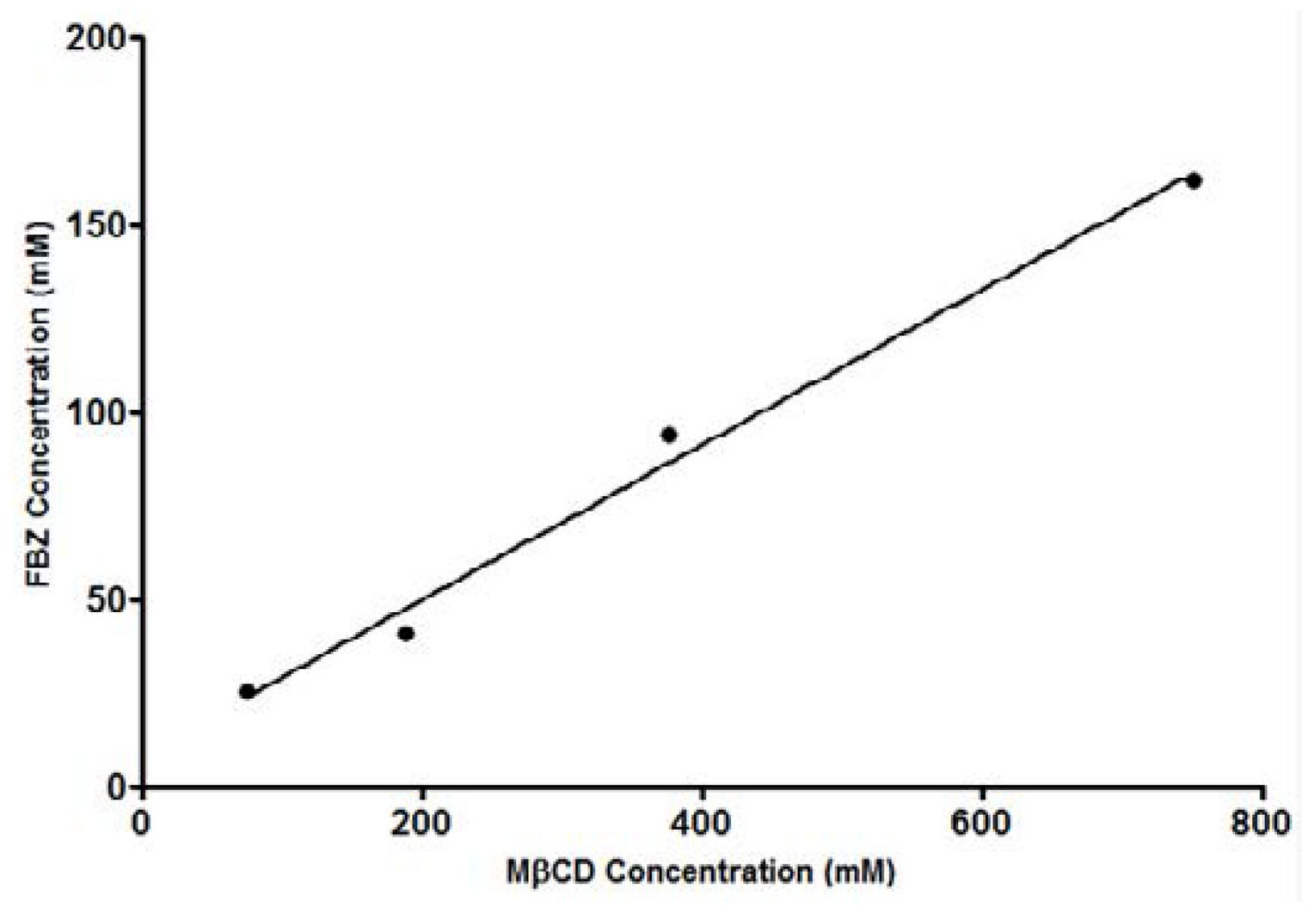
Effects of methyl-β-cyclodextrin (mβCD) on the solubility of FBZ.

Effects of mβCD on the aqueous solubility of FBZ was studied using the phase-solubility approach where FBZ was added to mβCD solution to determine the extent of FBZ solubility. From Figure 5 it was observed that solubility of FBZ increases as the concentration of cyclodextrin is increased showing a linear increase in solubility. This linear increase indicates a 1:1 complex formation with mβCD and the correlation coefficient of the linear regression of the phase solubility curve was than 0.99 indicating a good fit. The calculated complexation constant K_1:1_ of FBZ with mβCD was 0.00706 mM^−1^ using equation 3. In addition, the K_1:2_ complexation constant was calculated, based on equation 4, to be 8.98 × 10^−8^ mM^−1^ which was significantly lower than the K_1:1_ complex. The lower value of K_1:2_ compared with K_1:1_ indicates that the 1:1 complex was the predominant form.

### Release kinetics of FBZ from FBZ-mβCD nanoparticles

**Figure 6:**
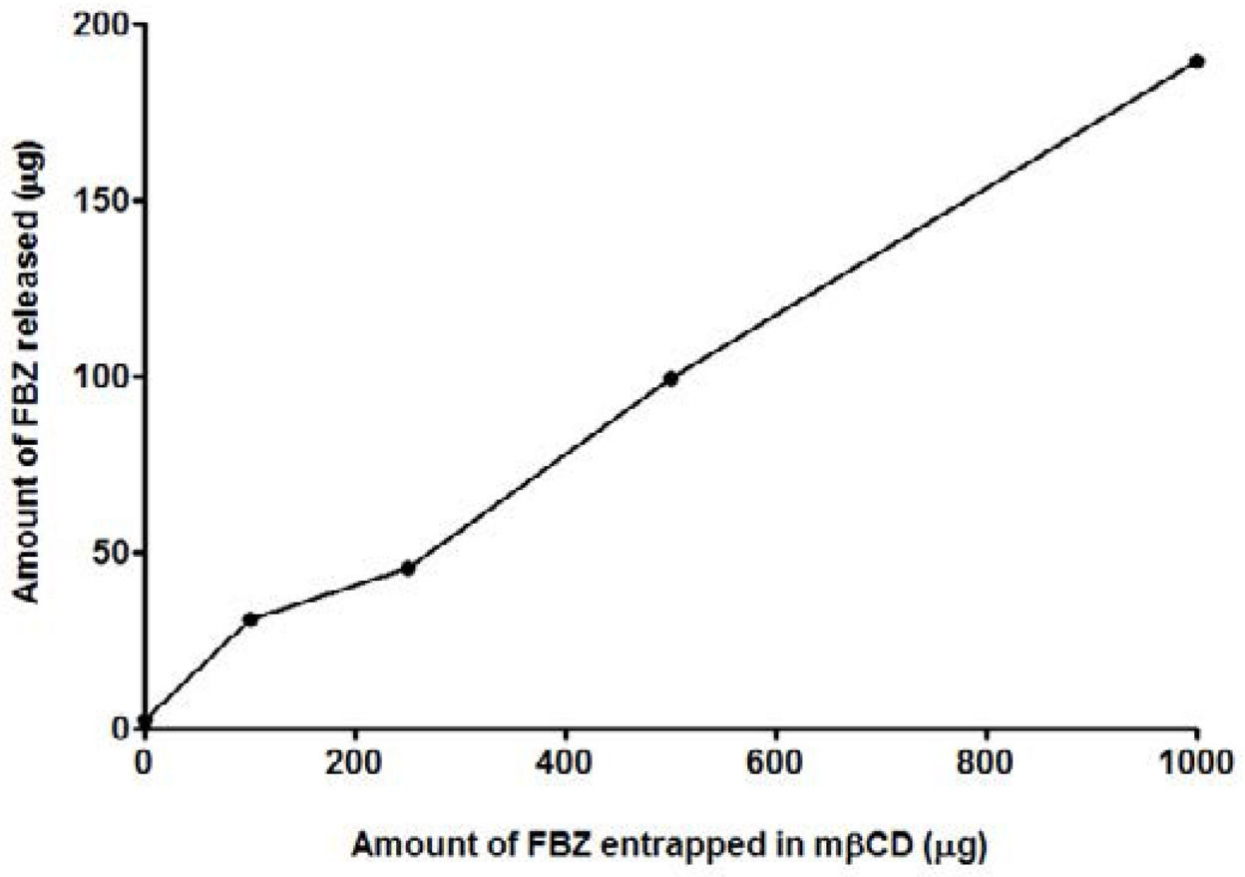
In-vitro release kinetics of FBZ loaded methyl-β-cyclodextrin (FBZ-mβCD) for a period of 24 hr at 37°C.

In-vitro release of FBZ was conducted and the results show a linear relation between the FBZ released and amount of mβCD used to entrap FBZ. From Figure 6, we observed that the higher the amount of mβCD employed in loading FBZ, the greater the released amount of FBZ. This agrees with the findings in Figure 5 where increasing amount of mβCD improves the solubility of FBZ.

### Characterization of FBZ-PLGA nanoparticles

Particle size and zeta potential FBZ-PLGAnps were determined as 216.20 ± 6.20 nm and −5.47 ± 0.83 mV while that of PLGAnps were 147.68 ± 18.07 nm and −2.92 ± 0.64 mV (Table 1). FBZ-PLGAnps were about 1.46 fold larger than PLGA nanoparticles is mostly likely due to FBZ entrapment. We also observed that zeta potential of FBZ-PLGAnps was 1.87 fold-high compared to that of PLGAnps. The negative charges exhibited by both nanoparticles are typical of PLGA polymers [24,26,27].

### MDCK cells viability and internalization of 125 μg of FBZ-mβCD nanoparticles

**Figure 7:**
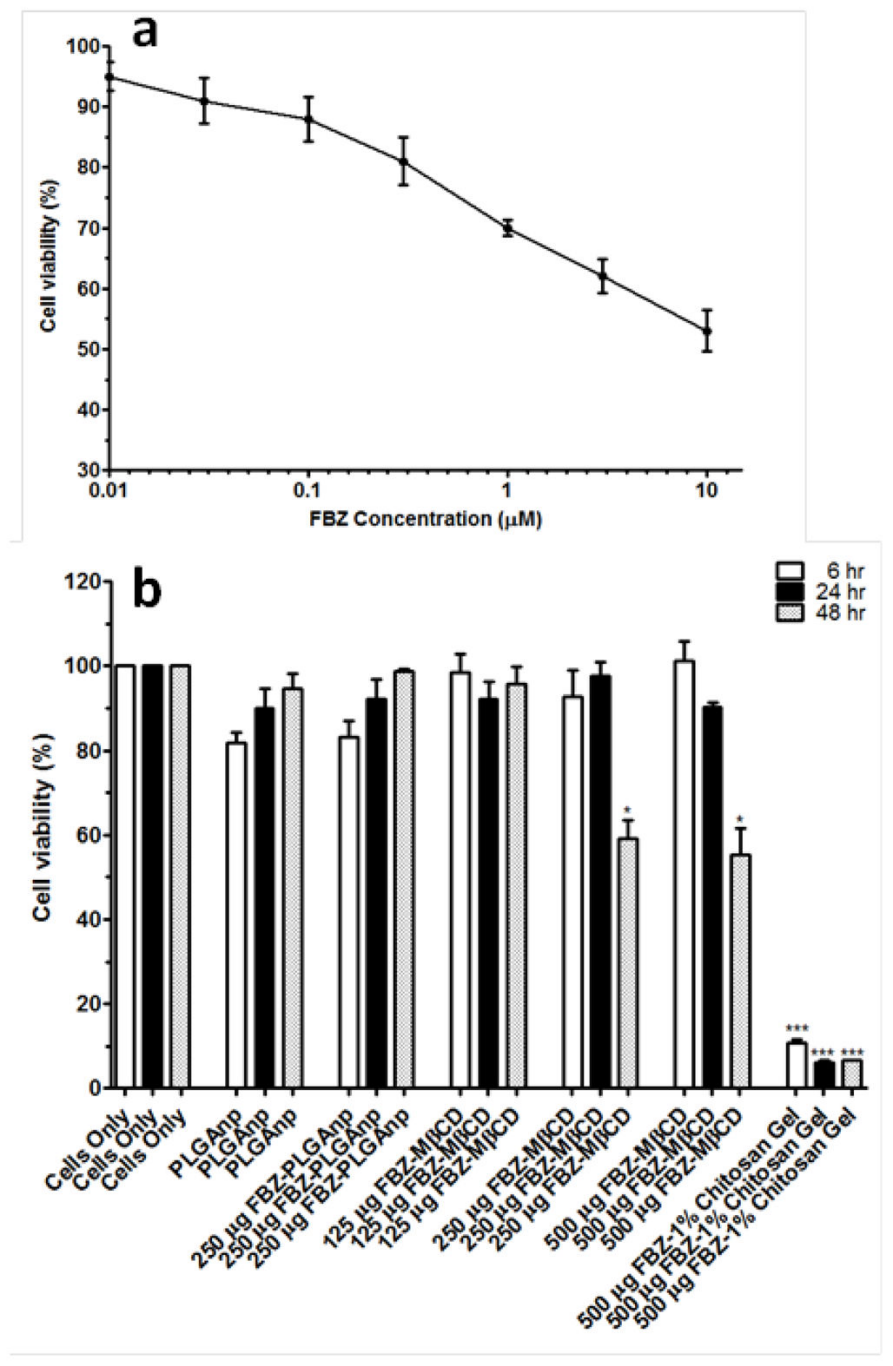
Percent viability of MDCK cells after cells were treated with increasing concentrations of FBZ for 48 hr (a), percent viability of MDCK cells when treated with PLGAnps, FBZ-PLGAnps, FBZ-mβCD and FBZ-chitosan nanoparticles at 6, 24 and 48 hr. Data are expressed as mean ± SEM (n=3). _*;_ p<0.05, _***;_ p<0.001.

**Figure 8:**
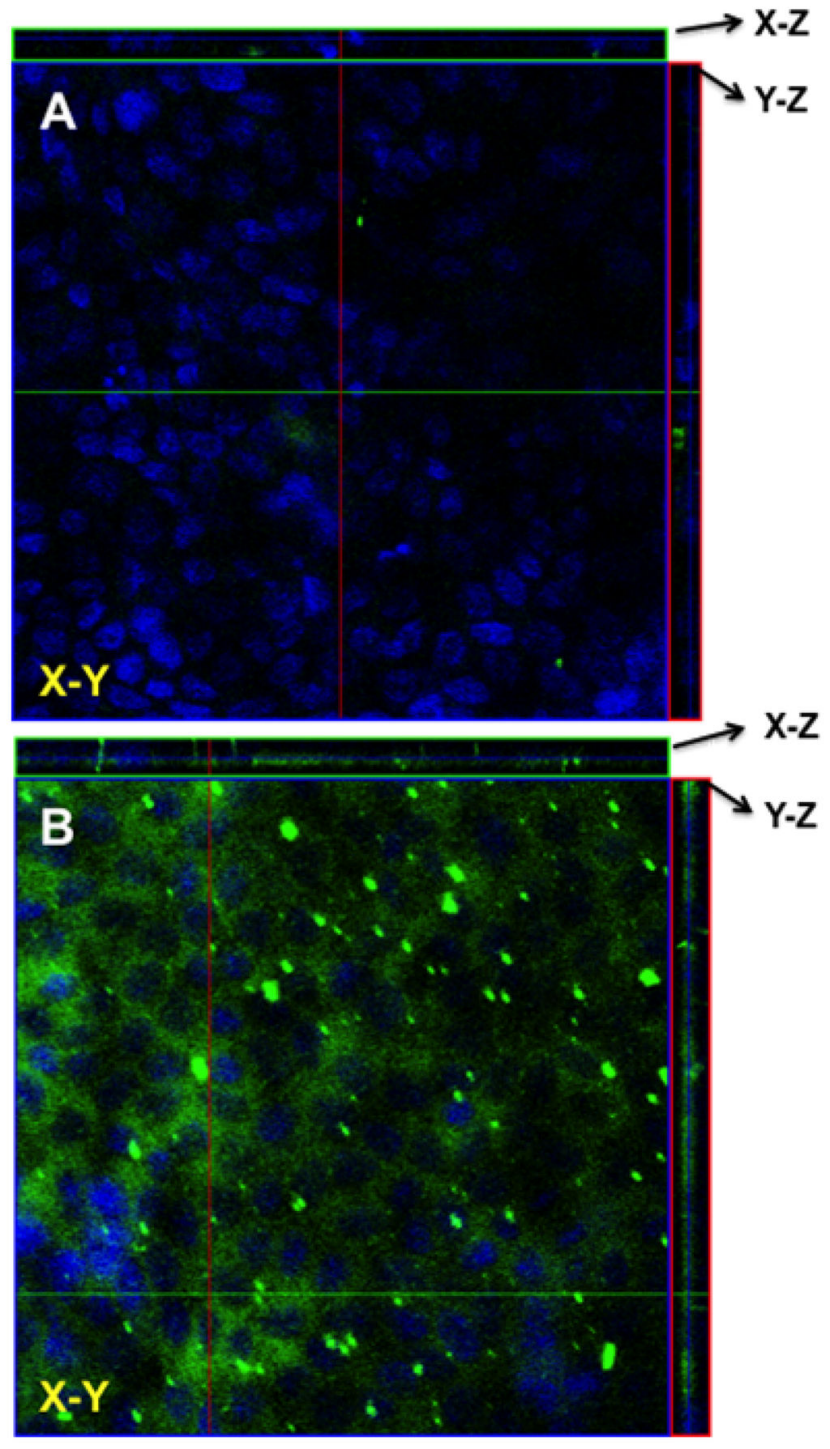
The uptake of (A) FBZ-cPLGAnps in MDCK cells as seen using laser confocal microscopy. The nanoparticles are depicted with green fluorescence whereas the cell nucleus is shown with blue fluorescence.

To evaluate the effect of various FBZ entrapped nanoparticles on MDCK cells, we first determined the effect of increasing FZB concentration on the MDCK cells viability. After 48 hr of exposure, we observed that 0.01 μM of FBZ barely have any effect on MDCK cells while 60% of MDCK cells were still viable after incubating them with 10 μm FBZ (Figure 7A). Figure 7B shows the graphical representation of the viability of MDCK cells when exposed to varying concentrations of various nanoparticles. The MDCK cells did not show any significant decrease in viability when treated with PLGAnps, FBZ-PLGAnps and 125 μg of FBZ-mβCDnps for the exposed period of 6, 12 or 48 hr. However, 250 μg of FBZ-mβCDnps and 500 μg of FBZ-mβCDnps have a significant decrease on MDCK cells’ viability (p<0.05) when they were exposed to the cells for 48 hr. Of all the formulations, only 500 μg FBZ-1% chitosan gel was cytotoxic on MDCK cells compared with the control. On the cellular uptake of nanoparticles, 125 μg of FBZ-mβCDnps was poorly internalized by the polarized MDCK cell monolayer. Only chitosan coated FBZ loaded-PLGAnps (FBZ-cPLGAnps), showed a significant accumulation in polarized MDCK cell monolayer as shown in Figure 8B, whereas, Figure 8A show the presence of nuclei in untreated MDCK cells.

## Discussion

Ftibamzone (FBZ), a thiosemicarbazone, is currently being used in Asia and in parts of Europe to treat genital herpes. However, its effectiveness against the herpes simplex virus is not realized to the fullest, because of its low solubility and inability to accumulate in the vaginal epithelial cells, which harbor the virus. In this study we have systematically investigated the physicochemical properties of FBZ and arrived at a rational formulation that could most effectively deliver FBZ to the site of viral proliferation.

FBZ has very low aqueous solubility at pH of vaginal cavity, which is around 4, and at the plasma pH of 7.4. It does not have appreciable solubility in biocompatible solvents such as ethanol or polyethylene glycol, but demonstrated significant solubility in DMSO and acetone which are unsuitable for pharmaceutical preparations. Our studies indicated that, beyond pH 10 the negative group gets more ionized, as a result the solubility of FBZ increases exponentially. Therefore, to increase FBZ solubility at pH 4, we employed mβCD that increased the solubility of lipophilic drugs by incorporating them in its lipophilic core. The mβCD forms a 1:1 with FBZ to a minor extent, but predominantly forms a 1:2 complex. However, the permeability and subsequent accumulation of FBZ-mβCD in polarized epithelial cell culture model was low. To circumvent such permeability challenges, we incorporated FBZ-mβCD complexes in bioadhesive and biocompatible chitosan gels, which are known to improve the drug permeability across the cellular barriers. However, these chitosan gel formulations compromised the integrity of epithelial cells or the host cells.

Hence, we designed FBZ entrapped PLGAnp (FBZ-PLGAnps) with a particle size of around 200 nm with a moderate negative charge density on the surface. When these nanoparticles were primed with 0.1% chitosan gel, the surface positive charges of the nanoparticles increased significantly. Despite increase in particle size to 280 ± 16 nm, the chitosan coated FBZ-PLGAnps were able to accumulate significantly in MDCK cells and had insignificantly low cytotoxicity compared with the control cells.

These results clearly demonstrate that solubility of FBZ could be significantly improved through the formation of FBZ-mβCD complexes. However, these complexes did not accumulate in the epithelial cells in appreciable quantities. But PLGAnps primed with chitosan serves an effective vehicle to carry FBZ to the MDCK cells where the herpes simplex virus resides and replicates.

The study demonstrates that FBZ-PLGAnp nanoparticles could significantly enhance the solubility and absorption of FBZ compared with FBZ alone and has the potential to be used as an effective delivery system to treat genital herpes. Further studies are needed to investigate and compare the antiviral activity of FBZ-PLGAnp and FBZ on herpes simplex virus.
